# Comparative Analysis of Two Real-Time PCR Techniques for the Detection of Congenital Syphilis in Umbilical Cord Blood

**DOI:** 10.3390/diagnostics16203408

**Published:** 2026-10-20

**Authors:** Gino Nicolas Algarra-Tavera, Angie Jimena Páramo-Cipagauta, Andrea Marisol Moreno-Gonzalez, Addy Cecilia Helguera-Repetto, Claudia Andrea Cruz-Baquero

**Affiliations:** 1 Facultad de Ciencias de la Salud, Universidad Colegio Mayor de Cundinamarca, Bogota 111711, Colombia; 2 Departamento de Inmunobioquímica, Instituto Nacional de Perinatología Isidro Espinosa de los Reyes, Ciudad de México 11000, Mexico

**Keywords:** congenital syphilis, real time PCR, umbilical cord blood, molecular diagnosis, *Treponema pallidum*, qPCR, TaqMan assay, SYBR Green, neonatal infection

## Abstract

**Background:** Syphilis remains a relevant public health problem and continues to be associated with adverse perinatal outcomes. In newborns, conventional serological tests are often difficult to interpret due to maternal antibody transfer and the immaturity of the neonatal immune response. These limitations have led to increasing interest in molecular approaches as complementary diagnostic tools. **Methods:** This pilot study compared two real-time PCR methodologies for detecting *Treponema pallidum* DNA in umbilical cord blood: a SYBR Green-based qPCR targeting the *polA* gene and a TaqMan probe-based qPCR targeting the *TpN47* gene. Thirty samples from neonates born to mothers with suspected gestational syphilis were analyzed under standardized conditions. Analytical performance and reproducibility were evaluated before application to clinical samples. **Results:** Both assays showed stable amplification and acceptable reproducibility. During standardization, the TaqMan assay reached a lower detection limit. However, when applied to clinical samples, the SYBR Green approach identified more positive cases (10/30 vs. 4/30), highlighting differences between analytical performance and behavior in clinical samples. **Conclusions:** Both methods were suitable for molecular detection of *T. pallidum*. However, their performance differed in neonatal samples, indicating that analytical sensitivity alone may not predict clinical detection capacity. Molecular testing in cord blood may provide complementary information when serological results are inconclusive.

## 1. Introduction

Syphilis is a sexually transmitted infection caused by *Treponema pallidum* subsp. *pallidum*, the etiological agent of syphilis, is a helical spirochete and an obligate human pathogen. Its study is challenging because of its physical transparency, poor staining properties (hence the term “pale spirochete”), microaerophilic nature, and inability to be cultured in vitro. These limitations in culturing and laboratory handling also make access to suitable controls relatively limited, posing an additional challenge for the standardization and validation of diagnostic methods [1,2,3]. Its relatively small genome comprises 1,138,006 base pairs and contains 1041 open reading frames (ORFs). In 2013, complete genome resequencing corrected approximately 11.5% of the errors identified in previously available sequences, affecting 119 genes, and revealed two distinct genetic subgroups circulating in humans. This genetic diversity is relevant when designing primers and probes, as sequence variation may affect hybridization and, consequently, molecular detection. During vertical transmission, the spirochete can cross the placenta and reach the fetal circulation through the umbilical cord, resulting in systemic fetal infection, with active multiplication in different organs and the development of local immune responses. Such infection may lead to severe outcomes, including miscarriage and stillbirth. However, conventional serological testing with RPR/VDRL at birth can be difficult to interpret, particularly because of the passive transplacental transfer of maternal IgG antibodies [3,4,5,6,7]. Quantitative real-time PCR (qPCR) performed on umbilical cord blood therefore represents an alternative approach for the direct and objective detection of the microorganism. Among the molecular targets previously validated for this purpose are the *polA* and *TpN47* genes. The *polA* gene encodes DNA polymerase I and contains a conserved region that has been used as a molecular target, with reported detection limits in blood ranging from 15 to 150 copies/mL. It has also been applied to strain typing and surveillance of macrolide resistance. The *TpN47* gene, in turn, encodes a 47-kDa immunogenic membrane lipoprotein and has been reported to have an analytical detection limit of 0.01–0.032 pg and 100% specificity, with no cross-reactivity with treponemes found in the normal flora [8]. The bacterial load detected through this target may also vary according to the clinical stage of the disease.

Congenital syphilis has increased substantially in recent years despite the long-standing availability of effective strategies for its prevention, diagnosis, and treatment [1,2,3,9,10]. This increase has been recognized as a public health emergency, particularly in settings where gaps in prenatal care persist [10]. Several factors may contribute to this trend, including inequitable access to healthcare services, particularly among underserved populations, limited awareness of syphilis among patients and healthcare providers, and difficulties in obtaining timely testing and treatment [10,11]. In some healthcare systems, limited availability of benzathine penicillin G has also affected timely treatment. Although a significant shortage of this medication has not been documented in Colombia, its availability remains a concern internationally, highlighting the importance of maintaining a reliable supply of benzathine penicillin G for the prevention of mother-to-child transmission [10].

Timely and complete treatment during pregnancy is essential to prevent mother-to-child transmission [12,13,14]. Qualitative studies have identified incomplete penicillin treatment and difficulties in accessing care as relevant barriers to congenital syphilis prevention, together with problems related to transportation, communication, appointment attendance, knowledge of the disease, and stigma [11]. In addition, intensified antibiotic regimens for early and late syphilis have been investigated, including the combination of benzathine penicillin G, ceftriaxone, and doxycycline, which was associated with a faster serological response and a higher proportion of cure than conventional treatment in the population studied [15]. These findings reinforce the importance of timely treatment and adequate follow-up during pregnancy to reduce the risk of vertical transmission.

This situation is especially relevant in Colombia, where recent national surveillance reports have shown a sustained increase in both gestational and congenital syphilis cases [16,17]. These trends suggest that, under routine clinical conditions, early detection and timely interruption of mother-to-child transmission are still not consistently achieved. In many cases, congenital syphilis is identified late or inferred indirectly, rather than confirmed during the early neonatal period.

One of the main challenges lies in neonatal diagnosis. Conventional serological tests, despite their widespread use, are difficult to interpret during the first hours of life [3,4,5,6,7]. The transplacental transfer of maternal IgG antibodies can lead to reactive results in uninfected newborns, while infected neonates may not yet have developed a detectable immune response. This creates a diagnostic gray zone, where clinical decisions often depend more on maternal history than on direct neonatal evidence.

Given these limitations, molecular methods have become increasingly relevant. Real-time polymerase chain reaction (qPCR) enables direct detection of *Treponema pallidum* DNA, independently of the host immune response, which may allow earlier and more objective diagnosis [4,18,19,20]. Among the molecular targets described, the polA and TpN47 genes have been widely used due to their conserved nature and prior validation in diagnostic assays [8,21].

However, the choice of amplification chemistry can influence performance. SYBR Green assays rely on intercalating dyes that bind to double-stranded DNA, which can increase sensitivity but also allow non-specific signals. In contrast, TaqMan assays use sequence-specific probes, generally providing greater specificity and clearer signal discrimination [19,22,23,24]. While these differences are well established at the analytical level, their impact may vary when applied to clinical samples.

Although PCR-based detection of *T. pallidum* has been evaluated in different biological specimens, direct comparisons between real-time PCR approaches in umbilical cord blood remain limited [18,25]. This is particularly true in Latin American hospital settings, where available data are still scarce. Umbilical cord blood represents an attractive sample because it can be obtained immediately after delivery without additional invasive procedures; however, its diagnostic value depends on how reliably molecular amplification performs in this biological matrix.

Beyond analytical parameters, the application of a molecular strategy using umbilical cord blood should also consider the reliability of the results and their potential clinical implications when making decisions for a newborn with suspected infection. In complex biological matrices, where the amount of treponemal DNA may be low, certain characteristics of the assay, such as signal specificity, the presence of nonspecific products, and potential interference from inhibitors, may affect diagnostic performance [19,22,23,24]. These limitations are particularly important during the neonatal period, since both false-positive and false-negative results may have relevant clinical consequences. A false-positive result could lead to unnecessary treatment with penicillin in an uninfected newborn, as well as an unnecessary hospitalization and iatrogenic separation of the mother and newborn. In contrast, a false-negative result could delay treatment in an infected newborn and increase the risk of severe neurological sequelae, multisystem organ involvement, or neonatal mortality. Therefore, comparing these qPCR strategies not only allows their analytical performance to be evaluated but also helps determine their usefulness for achieving more reliable detection of congenital syphilis.

Based on these considerations, we hypothesized that TaqMan probe-based qPCR could provide more consistent diagnostic performance than SYBR Green qPCR by reducing nonspecific signals and primer-dimer interference. Accordingly, this study compared SYBR Green qPCR targeting *polA* with TaqMan qPCR targeting *TpN47* to evaluate their analytical performance, reproducibility, and clinical detection capacity in umbilical cord blood from neonates born at a public hospital in Bogotá, Colombia.

## 2. Materials and Methods

### 2.1. Study Design and Setting

A pilot comparative laboratory-based study was carried out to assess the performance of two real-time PCR approaches for the molecular detection of congenital syphilis [19]. Umbilical cord blood samples were obtained from 30 neonates delivered at Hospital El Tunal, part of the Subred Integrada de Servicios de Salud Sur in Bogotá, Colombia, between 2023 and 2024.

The mothers were enrolled in the institution’s routine prenatal care programs. In this population, prenatal follow-up may be irregular or incomplete, and some women have contact with healthcare services mainly at the time of delivery, making timely diagnosis and follow-up of these infections challenging. Not all cases corresponded to confirmed gestational syphilis, which should be considered when interpreting the results. Nevertheless, the samples were included as part of a broader surveillance setting, in which early detection of potential vertical transmission remains relevant.

Because there is no definitive clinical gold standard at the time of delivery, the institution’s non-treponemal RPR serological test was used as a *relative reference standard* to categorize the 30 patient samples. Clinical sensitivity and specificity were not calculated for the cohort, as their estimation against an imperfect serological reference could lead to an inappropriate interpretation. Instead, clinical detection rates and diagnostic concordance between the methodologies were evaluated and compared.

### 2.2. Sample Collection

Umbilical cord blood was collected immediately after delivery under aseptic conditions. To strictly control pre-analytical variables, prevent enzymatic DNA degradation, and avoid PCR inhibition, approximately 3–4 mL of blood was drawn into EDTA tubes (lavender cap), explicitly avoiding heparin-based anticoagulants. Samples were transported to the laboratory under controlled refrigerated conditions (2–8 °C) and stored long-term at −20 °C until DNA extraction and molecular processing. This sample type was intentionally selected as it provides immediate access to neonatal biological material without requiring additional invasive procedures, making it particularly suitable for molecular analysis in the early postnatal period.

### 2.3. DNA Extraction and Processing

Genomic DNA was extracted from whole blood using the PureLink DNA Mini Kit (Thermo Fisher Scientific, Waltham, MA, USA), following the protocol previously standardized by the research group REMA [8,19]. DNA concentration and purity were measured using a NanoDrop Lite Plus spectrophotometer (Thermo Scientific) to ensure that the samples met minimum quality requirements for qPCR analysis. Although the extraction workflow was consistent, some variability is expected when working with cord blood. This type of sample may contain variable cellular content as well as substances that can interfere with PCR amplification. These factors were considered during data interpretation. All qPCR reactions were performed using a Heal Force Real-Time Thermal Cycler X960, provided by the Central Laboratory of Universidad Colegio Mayor de Cundinamarca, under previously established conditions.

### 2.4. SYBR Green Real Time PCR

The first approach consisted of a SYBR Green-based qPCR targeting the polA gene of Treponema pallidum. This gene encodes DNA polymerase I and has been widely used as a molecular target due to its relative conservation and consistent amplification performance. For standardization purposes, a recombinant plasmid carrying the *polA* insert cloned into the pJET1.2 blunt vector was used as a positive control (CloneJet PCR cloning kit, ThermoFisher Scientific, Waltham, MA 02451, USA). The construct was propagated in *Escherichia coli* and subsequently purified using a column-based approach (ZymoPURE express Plasmid MidiPrep, Zymo Research, Irvine, CA 92614, USA), providing a clean and stable DNA template for analytical evaluation. Reactions were carried out using the specific primers F: 5′TGATTGTGAAACGGATGGAT 3′ and R: 5′TTCCTGAAAGCAGATCGAAA 3′, under optimized thermal cycling conditions as follows: initial denaturation at 95 °C for 5 min, followed by 40 cycles of 95 °C for 15 s, 56 °C for 15 s and 72 °C for 15 s. Fluorescence was recorded after each amplification cycle and a melting curve analysis was subsequently performed by continuously monitoring fluorescence during a temperature ramp from 72 °C to 95 °C. Rather than relying solely on amplification curves, the melting profile was carefully examined to confirm that the detected signal corresponded to a unique and specific product and not to nonspecific amplification or primer-dimer formation. Serial dilutions of positive control DNA were used to construct analytical detection profiles and to evaluate assay performance under controlled conditions [19,22,23]. Reaction was performed using the Instrument Heal Force Real-Time Thermal Cycler X960.

All molecular reactions for each umbilical cord blood sample were conducted in duplicate to confirm technical reproducibility.

### 2.5. TaqMan Probe-Based Real Time PCR

The second methodology was based on a TaqMan probe targeting the TpN47 gene, which encodes a 47 kDa membrane lipoprotein commonly used in molecular detection of *T. pallidum* [8,21]. In this case, the positive control was not a synthetic construct, but DNA obtained from a rabbit testicular exudate infected with the Nichols strain of *Treponema pallidum*. This choice was intentional, as it allowed the assay to be evaluated using biological material that more closely resembles the complexity of clinical samples. This assay includes a sequence specific fluorescent probe in addition to forward and reverse primers, increasing specificity but also requiring proper hybridization conditions. Amplification was performed under standardized conditions, and fluorescence signals were monitored in a real time instrument Heal Force Real-Time Thermal Cycler X960. Serial dilutions of positive control DNA were used to construct analytical detection profiles and to evaluate assay performance under controlled conditions [22,24]. All molecular reactions for each umbilical cord blood sample were conducted in duplicate to confirm technical reproducibility.

### 2.6. Analytical Evaluation and Reproducibility

Both assays underwent a standardization phase that included repeated runs and serial dilutions of control DNA. This allowed assessment of amplification consistency, analytical sensitivity, and overall reproducibility. These conditions represent an analytical scenario and do not fully reflect the complexity of clinical samples. However, they provide a necessary baseline for understanding how each method behaves when technical variables are controlled.

### 2.7. Application to Clinical Samples

Following standardization, both qPCR methodologies were applied to the 30 DNA samples obtained from umbilical cord blood. Each sample was processed independently using both techniques. Results were interpreted qualitatively (positive or negative) based on amplification patterns and predefined criteria.

### 2.8. Statistical Analysis

Statistical analyses were performed using IBM SPSS Statistics, Version 27.0 (IBM Corp., Armonk, NY, USA). The qualitative results obtained with the SYBR Green and TaqMan assays were summarized as frequencies and percentages. Because both assays were applied to the same clinical samples and generated binary outcomes (positive/negative), paired results were compared using the exact McNemar test. Agreement between assays was assessed using Cohen’s kappa coefficient. A two-sided *p*-value < 0.05 was considered statistically significant.

### 2.9. Ethical Considerations

The study was conducted in accordance with the Declaration of Helsinki, and approved by the Institutional Review Board and Ethics Committee of Instituto Nacional de Perinatología Isidro Espinosa de los Reyes (protocol code 2024-1-44).

All samples were handled under confidentiality standards and in accordance with national regulations for research involving human-derived biological material.

## 3. Results

Out of the 30 samples analyzed, 16 showed serological reactivity and 14 were non-reactive according to the non-treponemal RPR test performed by the healthcare institution. To evaluate the correlation of qPCR detection with maternal-fetal antibody levels, molecular findings were stratified by RPR titer categories (Table 1).

The SYBR Green assay identified 10/30 samples (33.3%) as positive, whereas the TaqMan assay identified 4/30 (13.3%). Among the 30 paired samples, 2 were positive by both assays, 18 were negative by both, 8 were positive by SYBR Green but negative by TaqMan, and 2 were negative by SYBR Green but positive by TaqMan. The exact McNemar test showed no statistically significant difference in the paired proportions of positive results (*p* = 0.109). Overall agreement between the assays was 66.7%, with a Cohen’s kappa coefficient of 0.118, indicating low agreement beyond chance.

During the standardization phase, the SYBR Green qPCR targeting the polA gene showed a linear response across dilutions from 10^0^ to 10^−5^. At lower concentrations, amplification became less consistent, with signals appearing at later cycles, generally between 30 and 32. Based on this behavior, the detection limit was set at a Ct value of 30, corresponding to approximately 1 × 10^3^ fg/µL. The standard curve showed good linearity, with an R^2^ of 0.975 (Figure 1). The calculated amplification efficiency was 114.3%, slightly above the optimal range but still compatible with stable amplification. Melting curve analysis consistently revealed a single, well-defined peak, supporting the specificity of the amplified product.

When applied to clinical samples, the SYBR Green assay identified 10 positive cases (33.3%) and 20 negative cases (66.7%). The assay showed high reproducibility, with a coefficient of variation of 3.70%, indicating low variability between replicates (Figure 2).

In contrast, the TaqMan qPCR targeting the *TpN47* gene showed a broader linear dynamic range during standardization, extending from $10^{-1}$ to $10^{-7}$ dilutions. The analytical detection limit reached 0.01 fg/µL. The standard curve showed strong linearity, with an $R^{2}$ of 0.979. The calculated efficiency was 94.5%; this amplification efficiency allows for the reliable detection of low DNA concentrations. In silico analysis confirmed the specificity of both primers and probe (Figure 3).

When applied to the clinical samples, the TaqMan assay detected 4 positive cases (13.3%) and classified 26 samples (86.7%) as negative. The coefficient of variation was 3.04%, remaining within acceptable limits for molecular assays (Figure 4).

A clear difference was observed between both methodologies. The SYBR Green assay identified more than twice the number of positive samples compared to TaqMan. This finding contrasts with the analytical phase, where TaqMan showed a lower limit of detection. In other words, the method that appeared more sensitive under controlled conditions did not identify more positives when applied to clinical samples. However, because different control materials were used for the two assays, the resulting LoD estimates must be considered assay-specific and cannot be used for making direct comparison of analytical sensitivity between the two platforms. The analytical performance of each platform is shown in Table 2.

## 4. Discussion

The analytical LoD values obtained for the two assays should be interpreted cautiously, as different control materials were used for their determination. For the SYBR Green assay, the analytical LoD estimated using the recombinant rpoB plasmid was 1 × 10^3^ fg/µL, corresponding to approximately 3.0 × 10^2^ plasmid copies/µL. In contrast, for the TaqMan assay, the LoD was established using a *T. pallidum*-positive biological control containing both bacterial and rabbit DNA. Therefore, the reported concentration represents the DNA concentration of the biological control material and cannot be directly converted into *T. pallidum* genome equivalents. Consequently, the LoD values should not be interpreted as a direct comparison of the analytical sensitivity of the two assays. The lower apparent LoD observed for the TaqMan assay under its specific validation conditions is consistent with the greater target discrimination generally provided by probe-based detection; however, this finding should be interpreted in the context of the different control materials used for assay standardization [22,26,27]. From a purely analytical standpoint, there is little to question there. But that advantage did not fully translate into the neonatal samples.

Once umbilical cord blood was introduced, the behavior of both assays shifted. SYBR Green, despite having a higher detection limit during standardization, identified more positive samples. Not marginally more, more than twice as many. That difference forces a closer look. One possible explanation lies in the gap between analytical validation and clinical reality. Standard curves are built under ideal conditions: purified DNA, minimal interference, and stable targets [19,22]. Cord blood is something else entirely. Bacterial DNA is likely present in very low quantities, often mixed with large amounts of human DNA, along with proteins, heme compounds, and other elements that can interfere with amplification [4,18,20]. Under these conditions, performance tends to depend less on theoretical sensitivity and more on how the system tolerates complexity.

The higher positivity observed with the SYBR Green assay (10/30 vs. 4/30) represents a critical finding that warrants careful interpretation. This discrepancy may reflect two distinct possibilities: increased analytical sensitivity of the polA assay in complex matrices with low bacterial copy numbers, or potential false-positive results driven by non-specific amplification. Because intercalating dyes bind indiscriminately to all double-stranded DNA, non-specific binding can occasionally yield fluorescent signals. Although post-amplification melting curve analysis ($T_{m}$) was systematically applied to rule out primer-dimers and off-target amplicons, the intrinsic permissiveness of SYBR Green chemistry remains a potential contributing factor to the higher positivity rate. Integrating systematic amplicon sequencing will be essential for validating positive molecular results in future multi-center trials.

In that context, the chemistry itself begins to matter in a very practical way. SYBR Green is, by design, more permissive. It binds to any double-stranded DNA, so even low levels of amplification—sometimes close to the detection limit—can still produce a detectable signal [19,22]. TaqMan, on the other hand, requires a more coordinated process: both primers must anneal efficiently, and the probe must hybridize correctly for fluorescence to be released [26,27]. If any of those steps are affected, even slightly, the signal may drop below detection. This does not make TaqMan less specific, quite the opposite; but it does make it more demanding.

Pre-analytical factors (such as EDTA anticoagulant choice, transport delays, and prolonged storage) and cord blood matrix components, including fetal hemoglobin, heme, lactoferrin, and bilirubin can induce DNA degradation, inhibit polymerase activity, or cause fluorescence interference in intercalating dye assays like SYBR Green. Although silica-column DNA extraction was used to minimize protein and heme contaminants, potential residual matrix inhibition and pre-analytical variability must be considered when evaluating discordances between molecular platforms [8,19].

Taken together, these factors help explain why a method with superior analytical sensitivity did not yield higher positivity in clinical samples. That said, the results should not be interpreted as a competition where one method simply outperforms the other. Both assays were reproducible. Both were capable of detecting *T. pallidum* DNA. What differed was how each one responded when moved from controlled conditions to a biologically complex matrix.

From a clinical perspective, this distinction is relevant. One of the main challenges in congenital syphilis is the lack of reliable early diagnostic tools. Serology in neonates is often inconclusive in the first hours of life, and clinicians frequently rely on indirect evidence [3,7,9]. Molecular detection in cord blood does not replace existing diagnostic algorithms, but it may offer an additional layer of information at a time when decisions are often uncertain.

The diagnostic yield of qPCR in umbilical cord blood observed in our study should be contextualized within the broader landscape of molecular detection across different biological specimens in congenital syphilis [4,21]. Previous studies evaluating *T. pallidum* PCR performance have demonstrated varying sensitivity depending on the specimen type [4]. Placental tissue and amniotic fluid generally exhibit higher bacterial loads and molecular sensitivity during active fetal infection, as treponemes readily proliferate in vascularized fetal-maternal interfaces. Conversely, molecular testing in cerebrospinal fluid (CSF) is critical for identifying neurosyphilis but often yields lower sensitivity due to fluctuating spirochete densities [28]. Compared to peripheral neonatal blood, umbilical cord blood offers a non-invasive, immediately available matrix that eliminates pediatric venipuncture stress; however, its diagnostic performance can be affected by maternal blood contamination, local matrix inhibitors (such as fetal hemoglobin), or rapid clearance of spirochetemia [4,8]. Our findings of positive amplification in cord blood align with reported detection rates in neonatal blood (ranging from 25% to 45% in symptomatic or high-risk cohorts) [8], underscoring that cord blood qPCR serves as a viable, non-invasive surrogate for early direct pathogen detection when paired with clinical and serological evaluation.

A fundamental challenge in evaluating novel molecular assays for vertical transmission is the well-documented absence of a definitive clinical gold standard for congenital syphilis diagnosis in the immediate postnatal period. Standard neonatal serology is heavily confounded by the transplacental transfer of maternal IgG antibodies, which frequently yields reactive RPR/VDRL results in uninfected neonates, while early-stage infections or delayed immune responses may result in temporary seronegativity [3,4,5,6]. This diagnostic gap prevents the calculation of true clinical sensitivity and specificity within our patient cohort, representing a limitation in traditional performance evaluation. However, this exact limitation underscores the clinical necessity of direct molecular tools. Rather than attempting to match a flawed serological reference, the direct detection of *T. pallidum* DNA particularly through the highly restrictive, sequence-specific TaqMan chemistry provides an objective, independent marker of active treponemal dissemination. Therefore, discordant clinical results should not be interpreted merely as assay failures, but as complementary diagnostic dimensions.

Specifically, we discuss that while reactive RPR results are heavily confounded by maternal IgG transfer, direct qPCR detection (especially through sequence-specific TaqMan chemistry) provides an objective, independent marker of active treponemal dissemination, making discordant clinical results highly valuable complementary diagnostic dimensions rather than simple assay failures. Furthermore, direct microbiological confirmation methods—such as darkfield microscopy or Sanger sequencing of discordant cases—were not available under routine care conditions in this institutional setting. Consequently, direct qPCR should be interpreted as a valuable complementary diagnostic tool alongside institutional non-treponemal serology (RPR) rather than a standalone replacement.

An additional technical limitation of this pilot study was the omission of a simultaneous endogenous internal amplification control (e.g., human housekeeping gene) during clinical sample testing. Although the DNA extraction protocols and absence of matrix inhibition for these target genes were previously validated by our research group in umbilical cord blood matrices (Pinilla-Bermúdez et al., 2015 [8]), future large-scale prospective trials should systematically incorporate an internal control to continuously rule out sample-specific PCR inhibition.

Recent advances in molecular diagnostics for congenital syphilis have expanded beyond conventional real-time PCR to include emerging platforms such as isothermal amplification techniques (e.g., LAMP) [29] and digital PCR (dPCR) [2]. While isothermal methods provide rapid point-of-care potential [29], and dPCR offers absolute target quantification with enhanced resistance to matrix inhibitors [29], real-time PCR remains the most standardized and widely accessible quantitative molecular platform in clinical laboratories. Aligning with current CDC and WHO recommendations that emphasize the need for improved direct pathogen detection in neonates [30], our findings reinforce cord blood qPCR as a viable, non-invasive complementary tool within established clinical workflows.

From a public health perspective, comparing these platforms in umbilical cord blood presents a critical balance between economic viability and diagnostic patient safety. While the SYBR Green assay represents a lower-cost, more accessible alternative for resource-limited laboratories, its higher permissiveness in clinical samples necessitates rigorous melting curve analysis to prevent the risk of overtreatment driven by false positives. In contrast, the robustness of TaqMan chemistry prevents erroneous readings. Therefore, assay selection should not rely solely on analytical limits of detection but must be integrated into a clinical decision-making framework that considers local laboratory infrastructure and the imperative to guide immediate therapeutic decisions prior to hospital discharge. In particular, the sample size of 30 neonates restricts the statistical power of the study and increases the probability of a Type II error (failing to detect a true difference in clinical sensitivity between both qPCR assays). This recruitment limitation is a direct consequence of the complex socioeconomic and demographic vulnerabilities of the target population, characterized by late, highly fragmented, or absent prenatal care, which precluded continuous longitudinal follow-up during pregnancy. Due to these systemic public health barriers, this work should be characterized as a pilot study designed to establish the technical viability and immediate clinical utility of direct qPCR screening in umbilical cord blood in a real-world setting. Acknowledging these power constraints provides a strong rationale for future, large-scale investigations. The next phase of this research line is designed to significantly increase the sample size (n) through a multi-center approach, successfully enrolling a larger cohort of pregnant women with comprehensive, continuous clinical tracking spanning from early gestation through long-term neonatal outcomes. Still, data on molecular detection of congenital syphilis in cord blood remain limited, particularly in similar settings, and this study adds a useful comparative perspective.

Even a moderate increase in detection, if reliable, can be meaningful in this context. There are limitations to consider. The sample size was relatively small, and all samples were obtained from a single institution. Ct values were not available for a more detailed comparison of amplification dynamics, and complete correlation with neonatal clinical outcomes was not possible in all cases. Still, data on molecular detection of congenital syphilis in cord blood remain limited, particularly in similar settings, and this study adds a useful comparative perspective.

In the end, the findings point to something simple but important: analytical sensitivity alone does not define diagnostic performance. The nature of the sample, the stability of the DNA, the chosen target, and the chemistry of detection all interact in ways that are not always predictable. And in neonatal samples, this difference is of critical importance.

## 5. Conclusions

Although the TaqMan assay showed a lower apparent analytical limit of detection under its specific validation conditions, the SYBR Green assay identified a higher proportion of positive clinical samples, highlighting the influence of the biological matrix and assay configuration on molecular detection.

The observed differences cannot be attributed exclusively to the amplification chemistry, since the two assays targeted different genetic regions (*polA* and *TpN47*) and were standardized using different control materials. Therefore, the findings support the interpretation that both amplification chemistry and target selection may contribute to assay behavior in samples with potentially low concentrations of treponemal DNA and complex biological backgrounds.

From a clinical perspective, both approaches may provide complementary value, particularly in the early postnatal period when serological results can be difficult to interpret [3,7,9]. Molecular detection in umbilical cord blood, while not definitive on its own, may support earlier suspicion and contribute to more timely clinical decision-making.

Further studies with larger sample sizes and integrated clinical follow-up are needed to better define the role of these methodologies within diagnostic algorithms for congenital syphilis. Still, the present results suggest that selecting a qPCR strategy should involve not only analytical considerations but also an understanding of how each method performs under real clinical conditions.

## Figures and Tables

**Figure 1:**
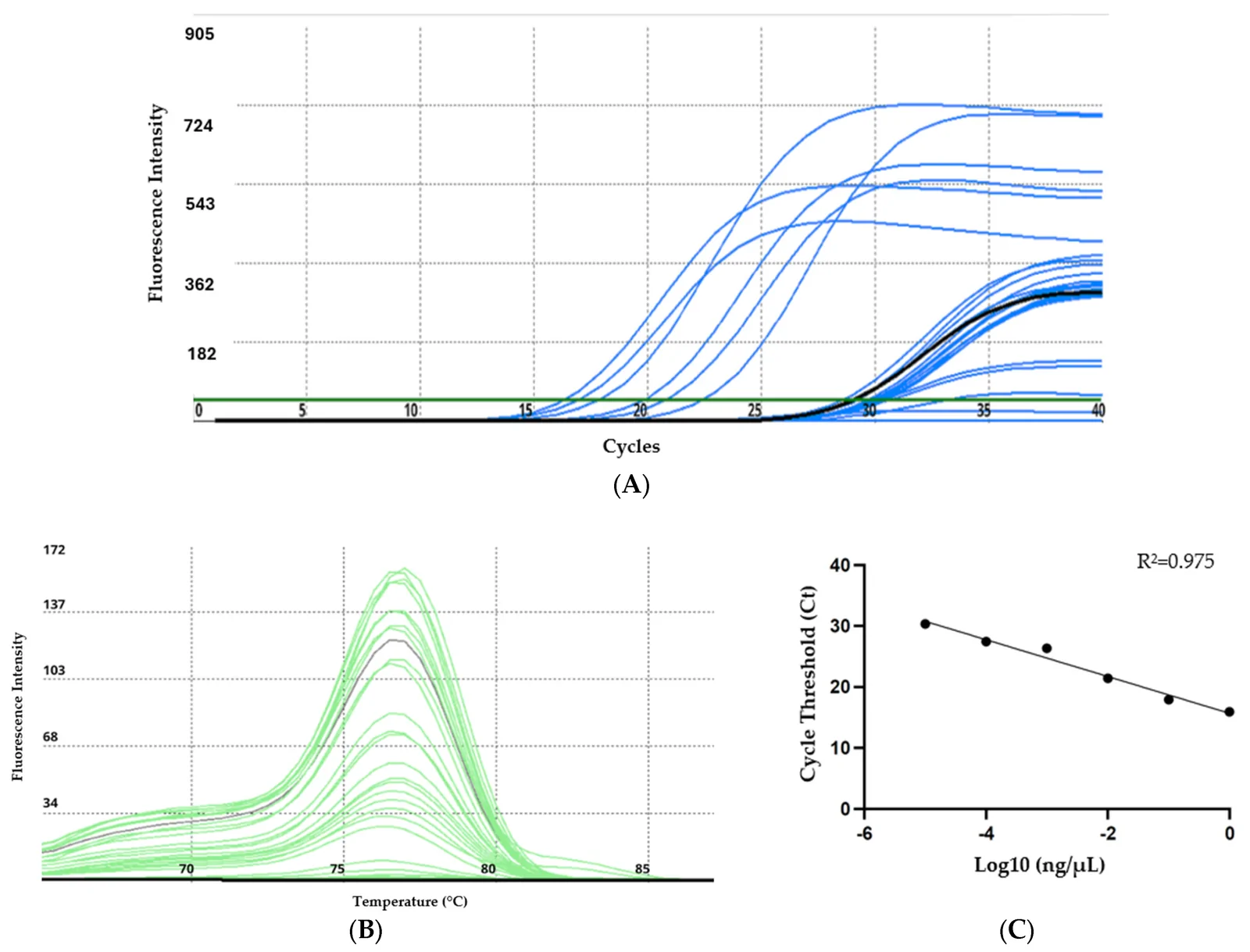
SYBR Green qPCR standardization analysis. (**A**) Amplification curves of duplicate serial dilutions. Blue lines represent amplification signals, the quantification cycle (Cq) threshold is shown in green, and the baseline in black. (**B**) Melting curve analysis of serial dilutions, shown as the negative derivative of fluorescence with respect to temperature. (**C**) Standard curve with serial dilutions plotted on the *x*-axis and Ct values on the *y*-axis. Each point corresponds to DNA concentration (ng/µL) and its associated Ct value. The linear regression equation and correlation coefficient (R^2^) are indicated.

**Figure 2:**
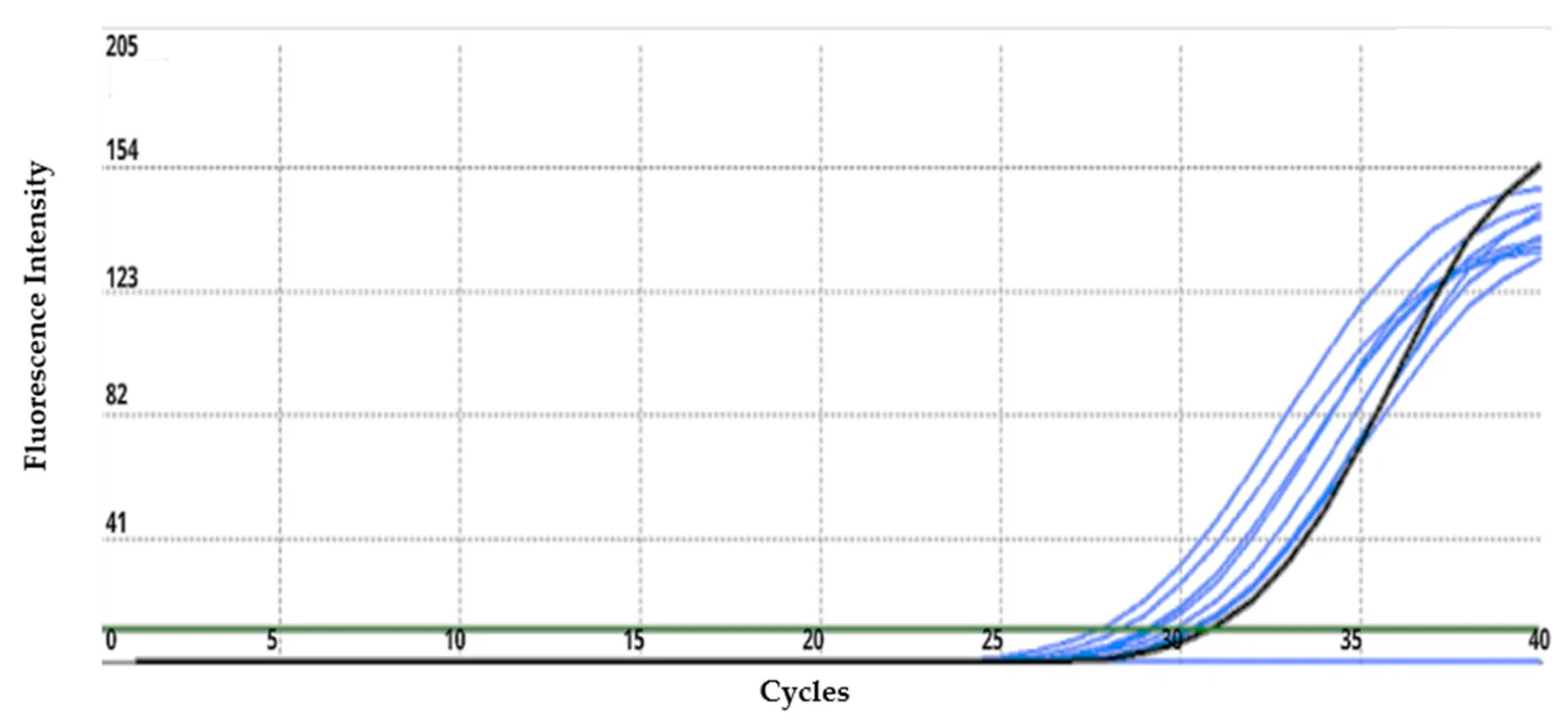
SYBR Green qPCR results of analyzed samples. Amplification curves of 15 samples processed by SYBR Green qPCR, shown as fluorescence versus amplification cycles (blue lines).

**Figure 3:**
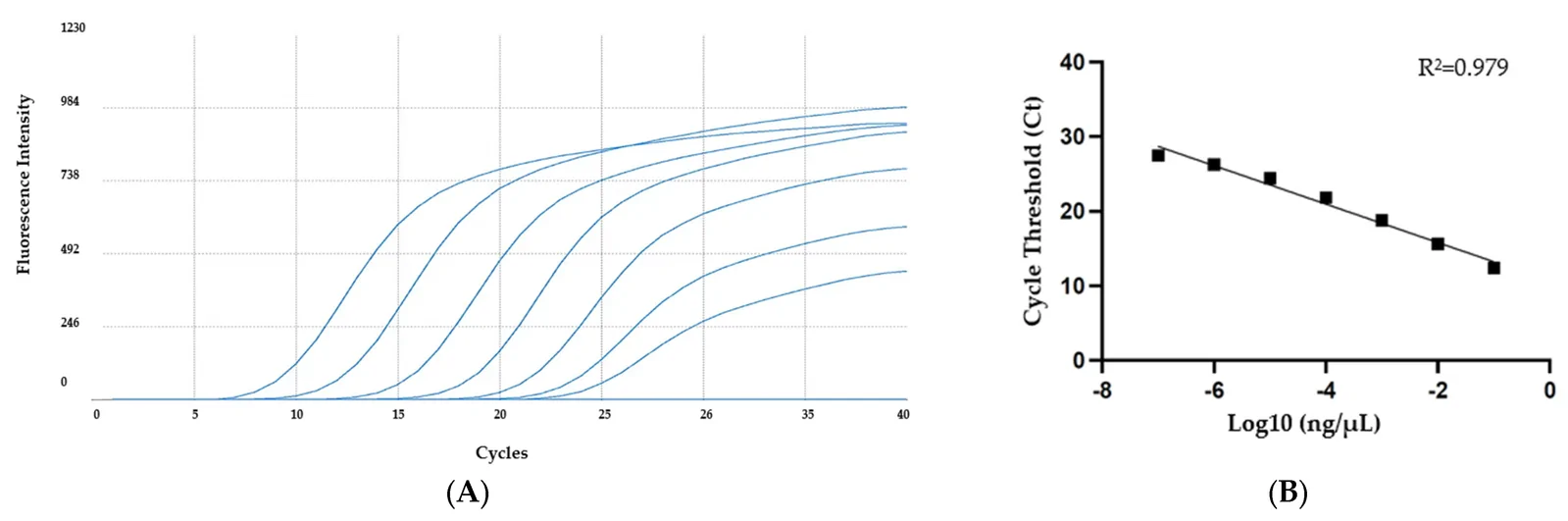
TaqMan probe-based qPCR standardization analysis. (**A**) Amplification curves of serial dilutions, shown as blue lines; the baseline is indicated in black. (**B**) Standard curve with serial dilutions on the *x*-axis and quantification cycle (Cq) values on the *y*-axis. Each point represents the corresponding DNA concentration (ng/µL) and Cq value. The linear regression equation and correlation coefficient (R^2^) are indicated.

**Figure 4:**
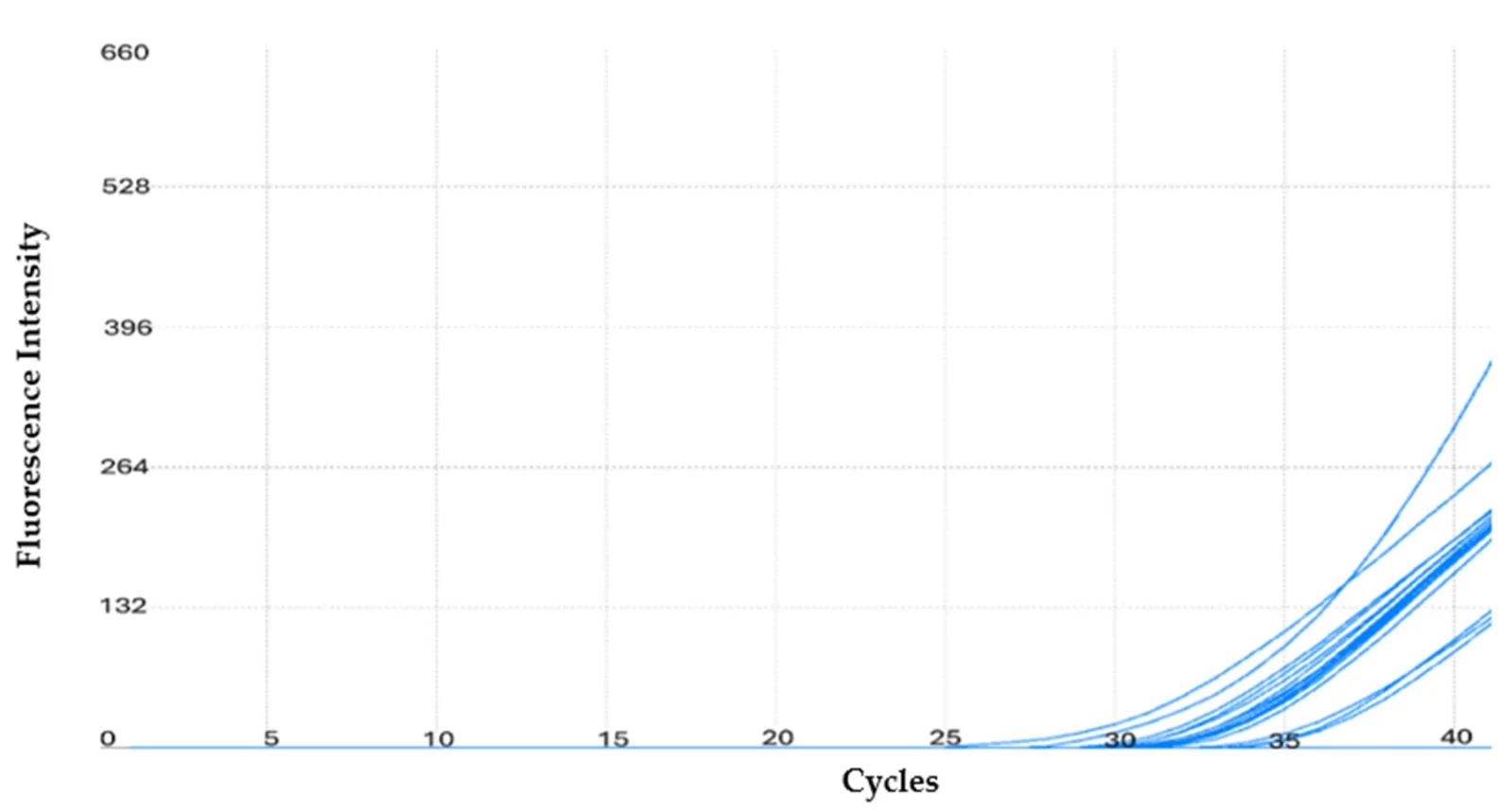
TaqMan probe-based qPCR results of analyzed samples. Amplification curves of samples processed by TaqMan probe-based qPCR, shown as fluorescence versus amplification cycles (blue lines).

**Table 1:** Diagnostic correlation between RPR serological titers and qPCR molecular detection (*N* = 30).

Sample (No.)	RPR Serology	qPCR SYBR Green (Ct)	qPCR SYBR Green Result	qPCR TaqMan Probe (Ct)	qPCR TaqMan Probe Result
1	Reactive 2 dils	30.88	Negative	32.50	Negative
2	Non-reactive	29.27	Positive	33.47	Negative
3	Non-reactive	27.73	Positive	26.57	Positive
4	Non-reactive	30.30	Negative	0.00	Negative
5	Reactive	29.64	Positive	33.28	Negative
6	Reactive 16 dils	28.83	Positive	30.23	Positive
7	Reactive 8 dils	28.16	Positive	0.00	Negative
8	Non-reactive	0.00	Negative	33.48	Negative
9	Non-reactive	30.12	Negative	0.00	Negative
10	Reactive 2 dils	30.10	Negative	0.00	Negative
11	Reactive 16 dils	31.44	Negative	0.00	Negative
12	Reactive 1 dils	29.00	Positive	35.60	Negative
13	Non-reactive	30.05	Negative	0.00	Negative
14	Non-reactive	29.19	Positive	0.00	Negative
15	Non-reactive	32.62	Negative	33.98	Negative
16	Reactive 8 dils	0.00	Negative	30.19	Positive
17	Reactive 2 dils	31.72	Negative	0.00	Negative
18	Reactive 4 dils	31.02	Negative	0.00	Negative
19	Reactive 2 dils	0.00	Negative	0.00	Negative
20	Reactive 2 dils	31.13	Negative	33.52	Negative
21	Non-reactive	29.37	Positive	33.25	Negative
22	Non-reactive	31.55	Negative	33.60	Negative
23	Non-reactive	0.00	Negative	33.08	Negative
24	Non-reactive	0.00	Negative	0.00	Negative
25	Non-reactive	0.00	Negative	0.00	Negative
26	Non-reactive	32.81	Negative	34.98	Negative
27	Reactive 2 dils	29.34	Positive	32.47	Negative
28	Reactive 2 dils	29.06	Positive	32.12	Negative
29	Reactive 8 dils	0.00	Negative	31.99	Positive
30	Reactive 2 dils	30.03	Negative	35.72	Negative

**Table 2:** Analytical performance comparison between SYBR Green and TaqMan qPCR assays.

Evaluated Parameter	SYBR Green qPCR	TaqMan Probe-Based qPCR
Analytical specificity	Validated by melting curve and in silico analysis	Validated by in silico analysis
Analytical sensitivity	1 × 10^3^ fg/µL (1000 fg/µL)	0.01 fg/µL
Correlation coefficient (R^2^)	0.9754	0.979
Efficiency	114.3%	94.5%
Coefficient of variation (CV)	3.70%	3.04%

## Data Availability

The data presented in this study are available from the corresponding author upon reasonable request. The datasets are not publicly available due to ethical and privacy restrictions related to patient information.

## Abbreviations

DNA	Deoxyribonucleic Acid
EDTA	Ethylenediaminetetraacetic Acid
INPer	Instituto Nacional de Perinatología Isidro Espinosa de los Reyes
qPCR	Quantitative Polymerase Chain Reaction
PCR	Polymerase Chain Reaction
RPR	Rapid Plasma Reagin
SYBR	SYBR Green
*T. pallidum*	*Treponema pallidum*
TpN47	*Treponema pallidum* 47-kDa Membrane Lipoprotein Gene
polA	DNA Polymerase I Gene of *Treponema pallidum*
Ct	Cycle Threshold
Cq	Quantification Cycle
CV	Coefficient of Variation
R^2^	Coefficient of Determination
UCMC	Universidad Colegio Mayor de Cundinamarca
WHO	World Health Organization
